# How Consumers and Physicians View New Medical Technology: Comparative Survey

**DOI:** 10.2196/jmir.4456

**Published:** 2015-09-14

**Authors:** Debra L Boeldt, Nathan E Wineinger, Jill Waalen, Shreya Gollamudi, Adam Grossberg, Steven R Steinhubl, Anna McCollister-Slipp, Marc A Rogers, Carey Silvers, Eric J Topol

**Affiliations:** ^1^ Scripps Translational Science Institute Scripps Health The Scripps Research Institute La Jolla, CA United States; ^2^ WebMD New York, NY United States

**Keywords:** digital revolution, healthcare, medical technology, physician and consumer attitudes, electronic health record, mobile health

## Abstract

**Background:**

As a result of the digital revolution coming to medicine, a number of new tools are becoming available and are starting to be introduced in clinical practice.

**Objective:**

We aim to assess health care professional and consumer attitudes toward new medical technology including smartphones, genetic testing, privacy, and patient-accessible electronic health records.

**Methods:**

We performed a survey with 1406 health care providers and 1102 consumer responders.

**Results:**

Consumers who completed the survey were more likely to prefer new technologies for a medical diagnosis (437/1102, 39.66%) compared with providers (194/1406, 13.80%; *P*<.001), with more providers (393/1406, 27.95%) than consumers (175/1102, 15.88%) reporting feeling uneasy about using technology for a diagnosis. Both providers and consumers supported genetic testing for various purposes, with providers (1234/1406, 87.77%) being significantly more likely than consumers (806/1102, 73.14%) to support genetic testing when planning to have a baby (*P*<.001). Similarly, 91.68% (1289/1406) of providers and 81.22% (895/1102) of consumers supported diagnosing problems in a fetus (*P*<.001). Among providers, 90.33% (1270/1406) were concerned that patients would experience anxiety after accessing health records, and 81.95% (1149/1406) felt it would lead to requests for unnecessary medical evaluations, but only 34.30% (378/1102; *P*<.001) and 24.59% (271/1102; *P*<.001) of consumers expressed the same concerns, respectively. Physicians (137/827, 16.6%) reported less concern about the use of technology for diagnosis compared to medical students (21/235, 8.9%; *P*=.03) and also more frequently felt that patients owned their medical record (323/827, 39.1%; and 30/235, 12.8%, respectively; *P*<.001).

**Conclusions:**

Consumers and health professionals differ significantly and broadly in their views of emerging medical technology, with more enthusiasm and support expressed by consumers.

## Introduction

As a result of the digital revolution coming to medicine, new tools are becoming available and are starting to be introduced in clinical practice, including genome sequencing and commercially available medical technologies, such as mobile phone-enabled self-monitoring of physiologic metrics or replacements of traditional laboratory tests. Many of these new digital tools raise questions about their impact on the patient-physician relationship, ethical standards, privacy, and security [1,2]. Yet there is limited widespread knowledge about the perceptions and support by consumers and health care professionals of these technologies. Assessment until now has been limited with respect to both scope and inclusion of views for both health care professionals and consumers. Understanding both patient and provider attitudes is essential if such technology is to be implemented in the future. Accordingly, we conducted a large-scale survey of the perceptions and comfort level towards new technologies by patients and providers by directing the same survey to both groups, adapted for each audience.

## Methods

### Study Participants and Data Collection

The technology survey assessed perspectives in two separate population samples, classified as either providers or consumers. A total of 21,812 health care professional members of Medscape who were active in the past year were invited via email to complete an online 15-item survey. Respondents completed the survey between August 22 and September 8, 2014. Lay WebMD website visitors from August 18-27, 2014, were invited via an interstitial invitation to complete a nearly identical online survey. This invitation was extended to total 456,243 consumers.

The Scripps Health Institutional Review Board reviewed and deemed this study exempt.

### Survey

Health care providers and consumers completed very similar 15-item surveys assessing attitudes toward new technology in medicine (see Multimedia Appendix 1). Participants also provided age and gender information. Consumers answered additional demographic questions, while providers reported their area of expertise and current work setting. To participate in the study, providers were categorized to one of the following occupations: doctor, nurse practitioner, physician assistant, nurse, or medical student. If the provider did not meet these criteria, their participation in the survey was terminated (n=144).

### New Technology

Health care providers and consumers responded to questions about the use of new medical technology for self-diagnosis of non–life-threatening conditions (see Multimedia Appendix 2, Q4). Respondents rated their willingness to use technology on a 3-point scale and rated whether they would use genetic testing for eight different medical scenarios. The use of mobile phones for conducting blood tests or submitting health information to a health care provider for four different conditions (eg, suspicious skin problem) was also evaluated.

### Privacy

Health care providers and consumers responded to one question assessing privacy and security concerns. The participants rated their level of reluctance to use digital technology due to concerns about privacy. More specifically, the question assessed levels of concern about storage, access, and sharing health records online, in addition to communicating electronically with health care providers.

### Medical Health Records

Four questions addressed attitudes towards electronic medical records. Providers and consumers rated whether patients should have access to lab results and doctor notes/procedures, or if doctors should share only information they deem appropriate. Moreover, consumers and health care providers identified ownership of a medical record, and whether access to medical records would cause patient anxiety, management of health, or unnecessary medical evaluations. Attitudes towards the immediacy of accessing lab test results were also assessed.

### Cost and Transparency

Three questions gauged likelihood to ask about medical costs prior to a procedure, patient rights to receive medical cost information prior to treatment, and access to prices charged by other providers. Providers were also asked whether they were willing to compete on the basis of price.

### Physical Exams and Imaging

Attitudes towards annual physical exams were evaluated with one question. Health care providers and consumers reported whether they felt an annual exam is necessary or whether there is interest in alternative forms of monitoring health. Additional concerns about exposure to radiation (eg, x-rays, mammograms, angiograms) were rated on a 7-point Likert scale in one question.

### Data Analysis

Age and gender differences between groups were assessed using chi-square statistics. Probit regression was conducted on survey items with categorical outcomes. Multinomial probit regression was used on 5 items to assess differences among multiple categorical (polytomous) outcomes. Linear regression was used for one item with a continuous outcome. For each survey item, all statistical analyses involving between-group comparisons were conducted accounting for age (continuous) and gender as covariates. Other covariates, though likely different between groups (eg, education, income), were unavailable. Significance results are presented without correction for multiple testing. All data analysis was conducted in R.

## Results

A total of 2508 surveys were completed, representing 1406 health care providers and 1102 consumers. Of the total number of Medscape members emailed (21,812), 6.4% of providers responded. A total of 456,243 consumers visiting the webpages owned and operated by WebMD were invited to participate in the survey. Health care provider respondents were younger than consumer respondents (mean age 45 versus 60 years respectively, *P*<.001) and included fewer females (providers: 704/1406, 50.07%; consumers: 776/1102, 70.42%; *P*<.001) (Table 1). Thus, all between-group comparisons are also presented accounting for age and gender covariates.

**Table 1:** Characteristics of providers and consumers.

Characteristics		Provider (N=1406), n (%)	Consumer (N=1102), n (%)	*P* value
Sex (% female)		704 (50.01)	776 (70.42)	<.001^a^
**Age in years**				<.001^a^
	20-29	228 (16.21)	40 (3.63)	
	30-39	323 (22.97)	41 (3.72)	
	40-49	311 (22.12)	119 (10.80)	
	50-59	332 (23.61)	296 (26.86)	
	60-69	175 (12.45)	373 (33.85)	
	70+	37 (3.63)	233 (21.14)	
**Politics**				
	Fiscally conservative, but socially liberal		159 (14.43)	
	Fiscally conservative, socially conservative		253 (22.96)	
	Fiscally liberal, socially conservative		24 (2.18)	
	Fiscally liberal, socially liberal		118 (10.71)	
	Middle of the road fiscally and socially		341 (30.94)	
	None of the above		207 (18.78)	
**Education**				
	Some high school		24 (2.18)	
	High school graduate		172 (15.61)	
	Some college		326 (29.58)	
	College (2 year)		115 (10.44)	
	College (4 year)		205 (18.60)	
	Postgraduate work		260 (23.59)	
**Marital status**				
	Married		616 (55.90)	
	Domestic partner		51 (4.62)	
	Never married		134 (12.16)	
	Divorced/separated		203 (18.42)	
	Widow		98 (8.89)	
**Income (USD)**				
	Under $16,000		106 (9.62)	
	$16,000-29,999		129 (11.71)	
	$30,000-44,999		173 (15.70)	
	$45,000-64,999		160 (14.52)	
	$65,000-79,999		119 (10.80)	
	$80,000-99,999		82 (7.44)	
	>$100,000		159 (14.43)	
	Declined to answer		174 (15.79)	
**Ethnicity**				
	African American/black		82 (7.44)	
	Caucasian/white		835 (75.77)	
	Hispanic (any)		52 (4.72)	
	Other		49 (4.45)	
	Declined to answer		84 (7.62)	

^a^Chi-square test.

Consumers were primarily college-educated with nearly one-quarter (260/1102) having some post-graduate training, 60.53% (667/1102) were married or had a domestic partner, and 75.77% (835/1102) were Caucasian.

The majority of health care providers were doctors (827/1406, 58.82%) with nurses representing the smallest group (85/1406, 6.05%) (Table 2). The most common physician specialties were family medicine (280/1406, 19.91%), internal medicine (224/1406, 15.93%), and pediatrics (168/1406, 11.95%).

**Table 2:** List of provider occupations, settings, and specialty (N=1406).

Provider characteristics		n (%)
**Occupation**		
	Doctor	827 (58.82)
	Medical student	235 (16.71)
	Nurse practitioner	152 (10.81)
	Physician assistant	107 (7.61)
	Nurse	85 (6.05)
**Primary practice setting**		
	Hospital	326 (23.19)
	Solo/group practice	311 (22.12)
	Outpatient clinic	190 (13.51)
	Academic, research, military, government	157 (11.17)
	Group practice owned by hospital	149 (10.60)
	Health care organization	131 (9.32)
**Specialty**		
	Family medicine	280 (19.91)
	Internal medicine	224 (15.93)
	Other specialty	231 (16.43)
	Pediatrics	168 (11.95)
	Other	153 (10.88)
	Psychiatry	67 (4.77)
	OB/GYN & women’s health	61 (4.34)
	General surgery	57 (4.05)
	Cardiology	47 (3.34)
	Neurology	47 (3.34)
	Emergency medicine	43 (3.06)
	Hematology/Oncology	28 (1.99)

### Technology

#### New Technology

Consumers were more likely to prefer using technology for self-diagnosis of non–life-threatening medical conditions (437/1102, 39.66%) compared with providers (194/1406, 13.80%), with more providers (393/1406, 27.95%) than consumers (175/1102, 15.88%) reporting feeling uneasy about consumers using technology for self-diagnosis. The majority of providers (819/1406, 58.25%) preferred a diagnosis be made by a professional compared with 44.46% (490/1102) among consumers (Table 3, Q1).

**Table 3:** Comparison of survey results between providers and consumers (relative risks [RR] in reference to providers:consumers).

Survey items		Provider, n (%)	Consumer, n (%)	RR	RR 95% CI	*P* value^d^
**Q1. Technology** ^a^ **(choose one)**						<.001
	Like technology, prefer professional diagnosis	819 (58.25)	490 (44.46)	1.3	1.2-1.4	
	Like technology for diagnosis	194 (13.80)	437 (39.66)	0.70	0.66-0.74	
	Uneasy using technology	393 (27.95)	175 (15.88)	1.2	1.1-1.2	
**Q2. Support genetic testing** ^b^ **(% No)**						
	Having a baby	172 (12.23)	296 (26.86)	0.83	0.80-0.87	<.001
	Diagnose problems in fetus	117 (8.32)	207 (18.78)	0.89	0.86-0.92	<.001
	Treat disease	39 (2.77)	70 (6.35)	0.96	0.95-0.98	<.001
	Disease prevention	80 (5.69)	73 (6.62)	0.99	0.97-1.0	.66
	Treat infections	228 (16.07)	122 (10.16)	1.1	1.0-1.1	<.001
	Identify drug side effects	172 (12.23)	145 (13.16)	0.99	0.96-1.0	.81
	Prolong lifespan	394 (28.02)	277 (25.14)	1.0	0.99-1.1	.05
	Identify cause of death	209 (14.86)	172 (15.61)	0.99	0.96-1.0	.56
Q3. Blood tests using smartphone^b^ (% No)		530 (37.70)	399(36.21)	1.0	0.96-1.1	.029
**Q4. Send/accept information via smartphone** ^b^ **(% No)**						
	Skin problem	737 (52.42)	412 (37.39)	1.3	1.2-1.4	<.001
	Heart rate/rhythm	554 (39.40)	379 (34.39)	1.1	1.0-1.1	<.001
	Eye exam	983 (69.91)	565 (51.27)	1.6	1.5-1.8	<.001
	Ear exam	962 (68.42)	509 (46.19)	1.7	1.6-1.9	<.001
Q5. Hesitant due to privacy concerns^b^ (% true)		492 (34.99)	466 (42.29)	.89	.83-.95	.033
**Q6. Ownership of medical record** ^a^ **(choose one)**						<.001
	Provider owns records	613 (43.60)	258 (23.41)	1.4	1.3-1.4	
	Patient owns records	431 (30.65)	594 (54.90)	0.66	0.62-0.71	
	Don’t know who owns records	362 (25.75)	250 (22.69)	1.0	0.99-1.1	
**Q7. Access to med records** ^b^ **(% I/Patient have/has a right to see)**						
	Patient has right to see all test results	1339 (95.23)	1060 (96.19)	0.80	0.55-1.2	.54
	Patient has right to see all doctors’ notes	884 (62.87)	984 (89.29)	0.29	0.24-0.35	<.001
**Q8. Access to EHR information** ^b^ **(% No)**						
	Could lead to feeling anxious about results	136 (9.67)	724 (65.70)	0.38	0.35-0.41	<.001
	Could lead to better management of my health	375 (26.67)	80 (7.26)	1.3	1.2-1.3	<.001
	Could lead to requesting unnecessary medical evaluations	257 (18.28)	831 (75.41)	0.30	0.28-0.33	<.001
**Q9. Access to lab tests** ^a^ **(choose one)**						<.001
	Provider should review	1096 (77.95)	641 (58.17)	1.9	1.7-2.1	
	Patients should have access	182 (12.94)	377 (34.21)	0.76	0.72-0.79	
	Doctors review results that may cause concern	128 (9.10)	84 (7.62)	1.0	0.99-1.0	
Q10. Cost medical procedure^b^ (% No)		718 (51.07)	549 (49.82)	1.0	0.94-1.1	.34
Q11. Right to know full cost of procedure^b^ (% agree)		1364 (97.01)	1057 (95.92)	1.4	0.90-2.1	.13
Q12. Prices charged by different providers^b^ (% No)		133 (9.46)	71 (6.44)	1.0	1.0-1.1	.40
**Q13. Annual physicals** ^a^ **(choose one)**						.039
	Annual exam is necessary	837 (59.53)	683 (61.98)	0.94	0.85-1.0	
	Alternatives for monitoring health	456 (32.43)	340 (30.85)	1.0	0.97-1.1	
	Annual physical unnecessary	113 (9.46)	79 (7.17)	1.0	0.99-1.0	
Q14. Concern about radiation exposure^c^, mean (SD)		4.28 (1.7)	3.53 (2.0)			<.001
**Q15. Feelings about new technology** ^b^ **(choose one)**						<.001
	Must be mastered	806 (57.33)	405 (36.75)	1.5	1.4-1.6	
	It is exciting	548 (38.98)	487 (44.19)	0.91	0.86-0.98	
	It is beyond me	37 (2.63)	166 (15.06)	0.87	0.85-0.90	
	It scares me	15 (1.07)	44 (3.99)	0.97	0.96-0.98	

^a^Multinomial probit regression.

^b^Probit regression.

^c^Linear regression.

^d^Age and gender modeled as covariates.

#### Genetic Testing

The majority of both providers and consumers supported genetic testing in medical situations, ranging from identifying and treating diseases such as cancer (providers: 1367/1406, 97.23%; consumers: 1032/1102, 93.65%) to prolonging lifespan (providers: 1012/1406, 71.98%; consumers: 825/1102, 74.86%). Providers and consumers similarly reported high support for using genetic testing for disease prevention, identifying drug side effects, and cause of death. Providers were significantly more likely than consumers to support the use of genetic testing when planning to have a baby (providers: 1234/1406, 87.77%; consumers: 806/1102, 73.14%) and diagnosing problems with a fetus (providers: 1289/1406, 91.68%; consumers: 895/1102, 81.22%). Consumers were more likely to support using genetic testing in treating infections (980/1102, 88.93%) than providers (1178/1406, 83.78%), although the absolute difference was not large (Table 3, Q2).

#### Smartphone Utilization

Health care providers were less likely to support the use of smartphones (*P*=.029) to perform blood tests. In contrast, consumers showed significantly greater support than providers for using smartphones for diagnosis of most of the surveyed conditions in place of an office visit, with 50-60% of consumers supporting smartphone delivery of information about skin problems, eye examinations, and ear examinations compared with 32-48% of providers (Table 3, Q4). Both providers (852/1406, 60.60%) and consumers (723/1102, 65.61%) endorsed using a smartphone to collect or provide heart rate information.

### Privacy

A substantial minority of both providers and consumers expressed hesitancy about privacy and security concerns when using digital health technology, although concern levels were significantly higher among consumers (466/1102, 42.29%) compared with providers (492/1406, 34.99%) (*P*=.033).

### Medical Health Records

#### Ownership

**Figure 1:**
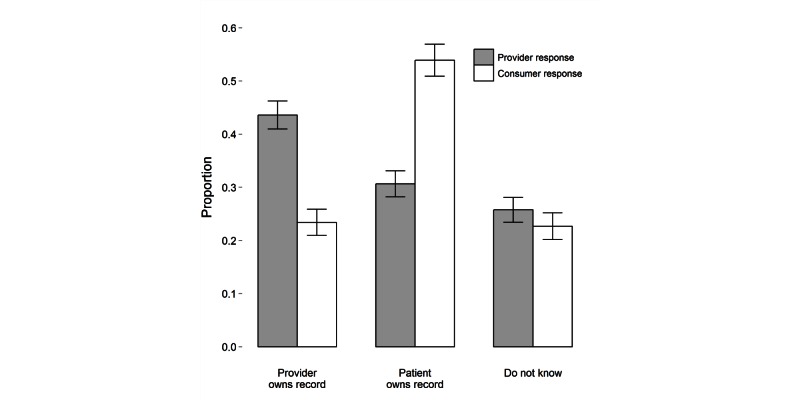
Proportion of responders who believed the patient or provider owned a patient’s medical record (error bars represent 95% confidence intervals).

When asked about ownership of the medical record, consumers and providers expressed significant differences in opinion: 43.60% (613/1406) of providers reported that they own their patients’ medical records, whereas 53.90% (594/1102) of consumers believed that patients own their medical record (see Figure 1 and Table 3, Q6). Approximately 20% of both groups responded that they did not know who owned the records.

#### Access to Medical Record Information

A high percentage of both providers (1339/1406, 95.23%) and consumers (1060/1102, 96.19%) agreed that patients should have a right to see all laboratory and diagnostic test results. In contrast, there was a significant difference in opinion regarding access to notes written by the doctor after visits or procedures, with 62.87% (884/1406) of providers agreeing that patients should have access to all notes compared with 89.29% (984/1102) of consumers (Table 3, Q7).

**Figure 2:**
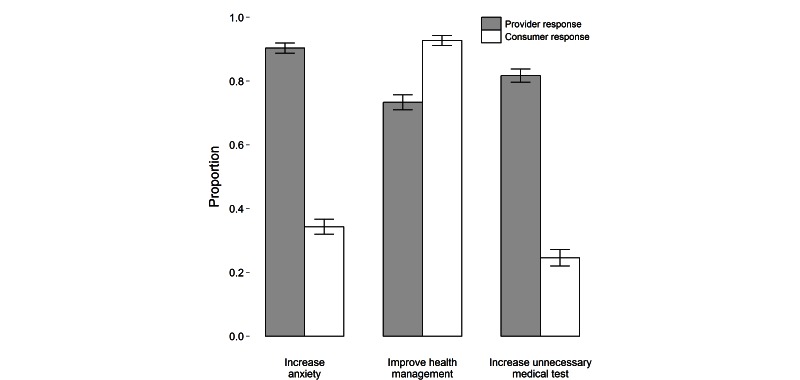
Proportion of responders who believed access to electronic health records would increase anxiety in patients, improve health management, or increase the number of unnecessary medical tests (error bars represent 95% confidence intervals).

Providers and consumers also differed in their beliefs regarding the consequences of patient access to detailed electronic health records (Figure 2). Most providers (1270/1406, 90.33%) reported concern that patients would experience anxiety after accessing health records, and 81.72% (1149/1406) felt it would lead to requests for unnecessary medical evaluations. Only 34.30% (378/1102) and 24.59% (271/1102) of consumers expressed the same concerns, respectively. While the majority of both groups agreed that access to records could lead to better management of the patient’s health, significantly fewer providers (1031/1406, 73.33%) than consumers (1022/1102, 92.74%) shared this belief (Figure 2).

When asked about access to lab tests, one-third of consumers (377/1102) agreed that patients should have access to all of their test results immediately compared to only 12.94% (182/1406) of providers, with a higher percentage of providers than consumers believing that doctors should review all lab results prior to sharing the information with patients (providers: 1096/1406, 77.95%; consumers: 641/1102, 58.17%). Fewer than 10% of individuals in both groups reported that doctors should review lab tests and determine which results may cause the patient worry before sharing the information.

### Cost and Transparency

Half of the respondents in both groups reported they (consumers) or their patients (providers) ask about the cost of medical services prior to pursuing treatment (Table 3, Q10). More than 90% of both groups agreed that patients should have the right to know the full cost prior to deciding to have a medical procedure and that patients should have access to prices charged by other providers (Table 3, Q11 and Q12).

### Physical Exams and Imaging

#### Physical Exams

Providers and consumers responded similarly to questions about physical exams. A majority of respondents reported that annual exams are necessary (providers: 837/1406, 59.53%; consumers: 683/1102, 61.98%). Less than 10% of both providers and consumers reported that annual exams are not necessary (Table 3, Q13).

#### Radiation Exposure

As shown in Table 3 (Q14), providers reported significantly more concern than consumers about patient exposure to radiation via various tests (eg, x-rays, mammograms, angiograms). On a 7-point scale, with higher scores corresponding to higher levels of concern, the mean concern level was 4.3 for providers and 3.5 for consumers.

#### Overall Opinion of New Technology

Providers and consumers differed significantly in their overall opinion of new technology (Table 3, Q15). In regard to overall feelings towards new technology, over half of the providers (806/1406, 57.33%) reported that it must be mastered to stay up-to-date compared with 36.75% (405/1102) of consumers. A higher percentage of consumers reported new technology is exciting (487/1102, 44.19%) compared with providers (548/1406, 38.98%). A subset of consumers reported that new technology is beyond them (166/1102, 15.06%) or scares them (44/1102, 3.99%), whereas, 2% or less of providers endorsed these responses.

#### Opinion Differences Among Health Care Providers

Differences in response to technology among physicians, medical students, and nurses, nurse practitioners, and physician assistants (collectively grouped as “nurses”) were also examined. Several differences emerged when comparing providers’ response to technology (Table 4). A higher proportion of physicians (137/827, 16.6%) preferred the use of technology for diagnosis when compared to medical students (21/235, 8.9%) and nurses (36/344, 10.5%). Similarly, medical students (154/235, 65.5%) and nurses (231/344, 67.2%) reported that they like technology but preferred that patients seek a professional diagnosis (physicians: 434/827, 52.5%; Table 4, Q1). In regard to genetic testing, physicians (581/827, 70.3%) and nurses (246/344, 71.5%) were less likely to support the use of genetic testing than medical students (185/235, 78.7%) for prolonging the lifespan (Q2). However, when using smartphones for diagnosis, medical students and nurses were less likely to accept an eye exam via a smartphone device (27%) when compared to physicians (274/827, 33.1%; Q4).

In terms of medical record ownership, a higher percentage of physicians (323/827, 39.1%) compared to nurses (78/344, 22.7%) and medical students (30/235, 12.8%) reported that the provider owned the medical record (Table 4, Q6). More doctors and nurses thought that patients should have access to doctors’ notes (physicians: 532/827, 64.3%; nurses: 235/344, 68.3%; medical students: 117/235, 49.8%; Q7). Although all groups agreed that access to electronic health records may increase patient anxiety, more physicians reported that access could lead to better management of health (physicians: 276/827, 33.4%; medical students: 42/235, 17.9%; nurses: 57/344, 16.6%). More nurses (90/344, 26.2%) thought access to electronic health records could lead to unnecessary medical evaluations than doctors or medical students (16%).

**Table 4:** Comparison of survey results among health care providers.

Survey items		Doctor, n (%)	Medical student, n (%)	Nurses^d^, n (%)	*P* value^e^
**Q1. Technology** ^a^ **(choose one)**					.03
	Like technology, prefer professional diagnosis	434 (52.5)	154 (65.5)	231 (67.2)	
	Like technology for diagnosis	137 (16.6)	21 (8.9)	36 (10.5)	
	Uneasy using technology	256 (31.0)	60 (25.5)	77 (22.4)	
**Q2. Support genetic testing** ^b^ **(% No)**					
	Having a baby	112 (13.5)	23 (9.8)	37 (10.8)	.58
	Diagnose problems in fetus	70 (8.5)	18 (7.7)	29 (8.4)	.89
	Treat disease	27 (3.3)	5 (2.1)	7 (2.0)	.98
	Disease prevention	56 (6.8)	8 (3.4)	16 (4.7)	.05
	Treat Infections	147 (17.8)	23 (9.8)	58 (16.9)	.07
	Identify drug side effects	102 (12.3)	21 (8.9)	49 (14.2)	.65
	Prolong lifespan	246 (29.7)	50 (21.3)	98 (28.5)	.007
	Identify cause of death	132 (16.0)	30 (12.8)	47 (13.7)	.63
Q3. Blood tests using smartphone^b^ (% No)		303 (36.6)	92 (39.1)	135 (39.2)	.22
**Q4. Send/accept information via smartphone** ^b^ **(% No)**					
	Skin problem	440 (53.2)	124 (52.8)	173 (50.3)	.18
	Heart rate/rhythm	321 (38.8)	91 (38.7)	142 (41.3)	.68
	Eye exam	562 (68.0)	171 (72.8)	250 (72.7)	.02
	Ear exam	553 (66.9)	161 (68.5)	248 (72.1)	.62
Q5. Hesitant due to privacy concerns^b^ (% true)		332 (40.1)	66 (28.1)	93 (27.0)	<.001
**Q6. Ownership of medical record** ^a^ **(choose one)**					
	Provider owns records	323 (39.1)	30 (12.8)	78 (22.7)	
	Patient owns records	314 (38.0)	128 (54.5)	171 (49.7)	
	Don’t know who owns records	190 (23.0)	77 (32.8)	95 (27.6)	
**Q7. Access to med records** ^b^ **(% I/Patient have/has a right to see)**					
	Patient has right to see all test results	790 (95.5)	223 (94.9)	326 (94.8)	.49
	Patient has right to see all doctors’ notes	532 (64.3)	117 (49.8)	235 (68.3)	.03
**Q8. Access to electronic health care record information** ^b^ **(% No)**					
	Could lead to feeling anxious about results	75 (9.1)	17 (7.2)	44 (12.8)	.25
	Could lead to better management of my health	276 (33.4)	42 (17.9)	57 (16.6)	<.001
	Could lead to requesting unnecessary medical evaluations	129 (15.6)	38 (16.2)	90 (26.2)	.02
**Q9. Access to lab tests** ^a^ **(choose one)**					.29
	Provider should review	639 (77.3)	181 (77.0)	276 (80.2)	
	Patients should have access	122 (14.8)	23 (9.8)	37 (10.8)	
	Doctors review results that may cause concern	66 (8.0)	31 (13.2)	31 (9.0)	
Q10. Cost medical procedure^b^ (% No)		421 (50.9)	125 (53.2)	172 (50.0)	.94
Q11. Right to know full cost of procedure^b^ (% agree)		803 (97.1)	228 (97.0)	333 (96.8)	.89
Q12. Prices charged by different providers^b^ (% No)		84 (10.2)	29 (12.3)	20 (5.8)	.16
Q12a. Prepared to compete on price^b^ (% No)		362 (43.8)	101 (43.0)	102 (29.7)	<.001
**Q13. Annual physicals** ^a^ **(choose one)**					.55
	Annual exam is necessary	479 (57.9)	148 (63.0)	210 (61.0)	
	Alternatives for monitoring health	265 (32.0)	76 (32.3)	115 (33.4)	
	Annual physical unnecessary	83 (10.0)	11 (4.7)	19 (5.5)	
Q14. Concern about radiation exposure^c^, mean (SD)		4.44 (1.7)	3.97 (1.6)	4.10 (1.6)	.003
**Q15. Feelings about new technology** ^b^ **(choose one)**					.31
	Must be mastered	478 (57.8)	128 (54.5)	200 (58.1)	
	It is exciting	315 (38.1)	99 (42.1)	134 (39.0)	
	It is beyond me	26 (3.1)	4 (1.7)	7 (2.0)	
	It scares me	8 (1.0)	4 (1.7)	3 (0.9)	

^a^Multinomial probit regression.

^b^Probit regression.

^c^Linear regression.

^d^Nurses, nurse practitioners, and physician assistants.

^d^Age and gender modeled as covariates.

## Discussion

### Principal Findings

The opinions of consumers and health care providers who completed this survey differ significantly in many scenarios when it comes to new medical technology. Nevertheless, interest in utilizing new technology does exist, with 40% of respondents reporting excitement about using new devices. Although respondents expressed some hesitancy (eg, access to medical records, ownership of records, transmitting information via smartphone, privacy), a majority of individuals from both groups were also in favor of using new medical technology.

A high percentage of consumers and providers agreed on the use of genetic testing to help prevent disease, identify side effects of certain medications, peri-partum diagnostics, and identify cause of death. A recent report of the opinion of parents towards genetic testing early post-partum reinforces our finding of strong consumer support [3].

Similar to consumers, providers believed that patients should have access to lab and diagnostic tests and cost transparency for procedures, but the same was not true for access to office medical notes. Although consumers were willing to use technology for self-diagnosis, providers reported a higher level of unease accepting this information.

### Comparison With Prior Research

The largest differences between consumers and providers emerged when assessing access to electronic health records. A marked disparity between health care providers and consumers was noted over concerns that patients would experience an increase in anxiety and request unnecessary health care resources. In contrast to all providers, consumers believed that access would not lead to anxiety, but instead, result in better management of their health care. These perceptions mirror the results of the Open Notes study that found that patients do benefit from access to their medical notes and, although doctors anticipated negative psychological impact, few patients experienced symptoms of anxiety [4]. Prior research suggests that patient access to information generated by new technologies, such as genetic risk information, does not result in an increase in health care utilization [5]. Moreover, there is empirical support for the efficacy of electronic health records access [6] and, in general, patients respond positively to the information [1]. Nonetheless, there is a continued concern that more information via technology will burden physicians and medical resources [2] and that this may have an impact on confidentiality and privacy [1].

Providers were, for the most part, less willing to accept diagnostic information from their patient via smartphone, although that was somewhat information-type dependent with heart rhythm detection being twice as acceptable as diagnostic imaging. The use of camera phones provides another venue of communication, can be a form of empowerment, and can engage the patient in both the diagnostic and management of their own health. Furthermore, instead of hindering rapport, the additional communication and involvement could potentially lead to a stronger doctor-patient relationship [7].

While consumers expressed more privacy concerns for new technology than providers, it was surprising that less than half of the respondents expressed any security concerns. This contrasts with the results of a recent survey of just over 2000 individuals from the Office of the National Coordinator that found nearly 70% of respondents whose providers used electronic health records to be very or somewhat concerned about data privacy, and approximately 75% were concerned about data security [8]. This difference may reflect a higher level of trust in digital data by individuals who routinely used Web-based resources such as WebMD.

The ownership of a medical record was also an area of substantial divergence. Providers believed that they owned their patient’s medical record nearly twice as often as did consumers. In contrast, just over 50% of consumers believed that the patient owned their own record. Perhaps surprisingly, a higher proportion of doctors, when compared to other medical students, reported that the patient owned the medical record. Interestingly, a quarter of all respondents did not know who owned the medical record. Another surprising finding was that medical students tended to express more conservative views regarding use of technology in several areas compared with physicians, being more likely to prefer diagnoses to be made by health care providers and less likely to consider patients to own their medical records and to endorse patient access to provider notes.

### Limitations

Respondents represent a small proportion of Medscape members and WebMD consumers that elected to participate. Therefore, the results may not represent the larger population of medical providers and consumers. Furthermore, only about 6% of Medscape members and 1% of WebMD consumers who were offered this survey elected to complete it. Thus, our results should be interpreted within the context of two potential biases: (1) membership/visits to these corresponding websites, and (2) a small proportion of eligible respondents. However, a recent report of the results of two non-simultaneous surveys of consumer and provider opinions around digital technology in health care found results consistent with ours [9]. Future studies will benefit from collecting in-depth descriptive statistics and diverse samples to further understand nuanced differences between consumers and providers.

### Conclusions

Clinical validation of new digital technologies, with assessment of efficacy, safety, and cost-effectiveness, will be an important part of future research efforts. But understanding the attitudes of patients and physicians may be particularly useful before such validation can occur and especially prior to any widespread potential clinical implementation. The new technologies exemplify the disruption of existing systems of health care—medical information flowing directly to patients, such as with smartphone sensors and lab testing, or with the newfound ability for consumers to perform elements of the physical examination. Our results show that both consumers and health care professionals are generally supportive of these technologies, albeit with sizably greater support and enthusiasm among consumers. Furthermore, the sensitive issue of ownership and access to medical records, where a large gap between consumer and provider expectations exists despite recent clinical validation of transparency, requires considerable further attention. As medicine gets increasingly digitized, the forces favoring democratization will likely be intensified.

## Appendix

Multimedia Appendix 1 Survey items (consumer survey version).

Multimedia Appendix 2 Survey items (provider survey version).
