# Figure Correction: How Consumers and Physicians View New Medical Technology: Comparative Survey

**DOI:** 10.2196/jmir.5150

**Published:** 2015-12-18

**Authors:** Debra L Boeldt, Nathan E Wineinger, Jill Waalen, Shreya Gollamudi, Adam Grossberg, Steven R Steinhubl, Anna McCollister-Slipp, Marc A Rogers, Carey Silvers, Eric J Topol

**Affiliations:** ^1^ Scripps Translational Science Institute Scripps Health The Scripps Research Institute La Jolla, CA United States; ^2^ WebMD New York, NY United States

In Figure 2 of the paper “How Consumers and Physicians View New Medical Technology: Comparative Survey” (J Med Internet Res 2015;17[9]:e215), authors erroneously inverted the bars indicating the proportion of people who believed access to electronic health records would increase anxiety in patients, improve health management, or increase the number of unnecessary medical tests. The originally published paper showed the proportion of respondents who answered “No” to the question. The corrected figure now displays the proportion of people who responded “Yes.” The online version of this JMIR paper has been updated with this figure, and a corrected version was sent to PubMed Central.

**Figure 2:**
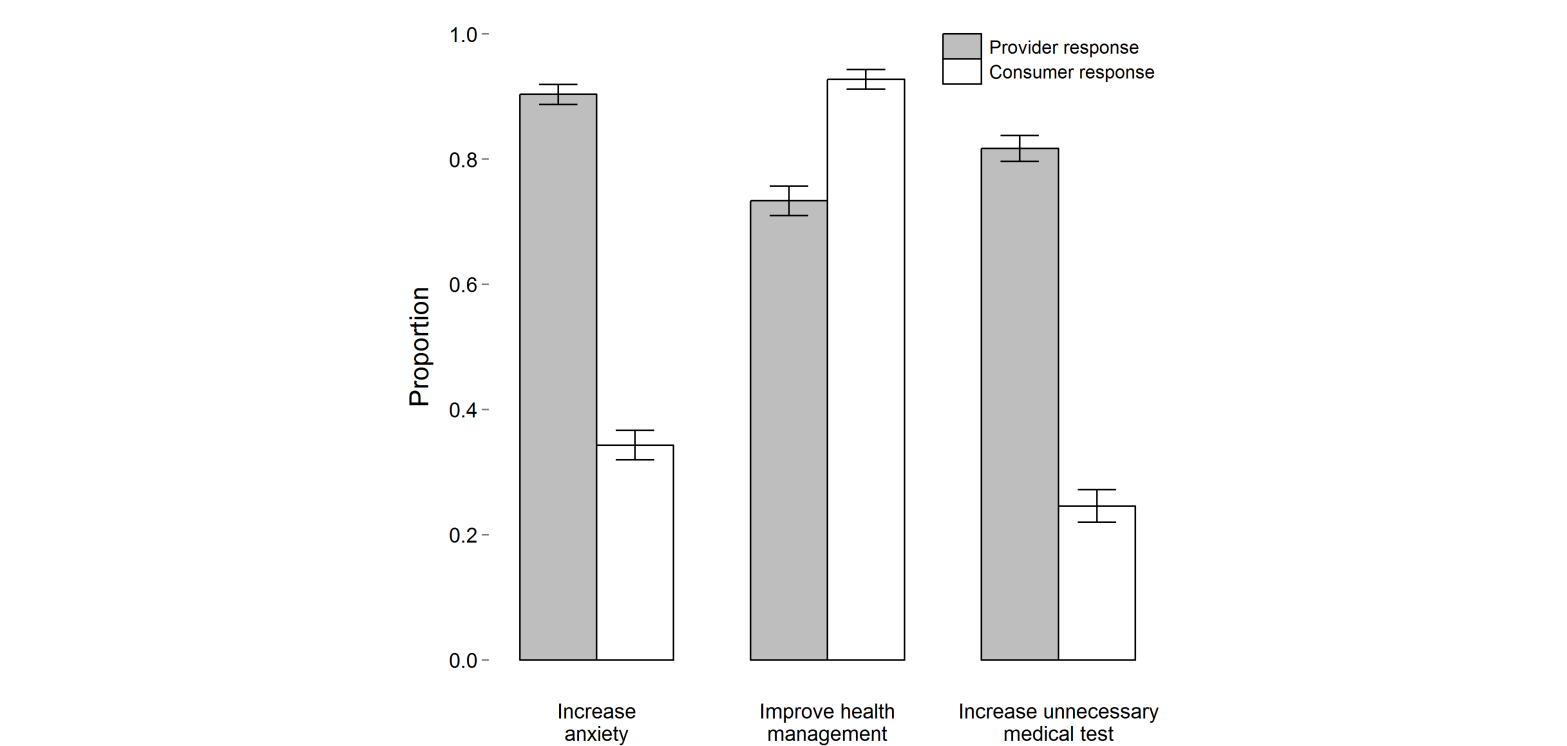
Proportion of responders who believed access to electronic health records would increase anxiety in patients, improve health management, or increase the number of unnecessary medical tests (error bars represent 95% confidence intervals).

