# Supplementary material for: How Consumers and Physicians View New Medical Technology: Comparative Survey
Source: J Med Internet Res. 2015 Sep 14;17(9):e215. doi: 10.2196/jmir.4456 (PMC4642377; doi:10.2196/jmir.4456)
Supplement: Multimedia Appendix 1 [file jmir_v17i9e215_app1.pdf]

1. Survey Items (Consumer Survey Version)

Q1. Suppose medical technology advanced to the point where you could test yourself and receive a diagnosis for non-life-threatening medical conditions. Which of the following statements best reflects how you would choose to use this new technology?

1. I would like to use this new technology for diagnosis
2. I would like to use this new technology to get tests done, but prefer that the diagnosis be made by a qualified healthcare professional
3. I am uneasy about using such technology and prefer an in-person examination/diagnosis by a healthcare professional

Q2. Do you agree with the use of genetic testing for each of the following medical situations? (YES/NO)

1. When planning to have a baby
2. To diagnose problems in a fetus
3. To identify and treat diseases (e.g. cancer)
4. For disease prevention
5. To identify and treat infections
6. To identify drug side effects
7. To prolong lifespan
8. To identify cause of death

Q3. In the future, it may be possible for you to perform blood tests using your smartphone. Would you be willing to do your own blood test using your smartphone? (YES/NO)

Q4. Would you be willing to use your smartphone to submit information to your healthcare professional about any of the following in order to replace an office visit: (YES/NO)

1. Suspicious skin problem
2. Heart rate and rhythm
3. Eye examination
4. Ear examination

Q5. Is the following statement true for you or not?
Because of concerns about privacy and security, I am hesitant to use digital health technology, such as storing, accessing, or sharing health records online and communicating with healthcare professionals electronically.
(TRUE FOR ME/NOT TRUE FOR ME)

Q6. Who owns your medical records?

1. I own them
2. My doctor or healthcare professionals own them
3. Don't know

Q7. Which of the following best represents your view toward accessing your electronic medical records?

1. Lab and diagnostic tests results (X-rays, etc.)
    a. I have a right to see all of them
    b. Doctors should share with me only what they think appropriate

2. Notes the doctor writes after visits or procedures
    a. I have a right to see all of them
    b. Doctors should share with me only what they think appropriate

Q8. Do you believe that having access to your detailed electronic health records could lead you to the following? (YES/NO)

1. Feeling anxious about the results
2. Better management of my health
3. Requesting unnecessary medical evaluations

Q9. Regarding lab test results, which do you think is the most appropriate?

1. Patients should have access to <u>all</u> their test results immediately
2. Doctors should review <u>all</u> test results before sharing with patients in case a discussion is needed
3. Doctors should review <u>only the types of test results that could cause patients to worry or panic</u> before sharing with patients

Q10. Do you tend to ask your healthcare providers questions about the cost of medical services prior to pursuing a course of treatment? (YES/NO)

Q11. Patients should have the right to know the full cost of a medical procedure before they decide whether to have it? (AGREE/DISAGREE)

Q12. Should patients have access to the prices charged by different providers for a medical procedure so they can shop around? (YES/NO)

Q12a. [PROVIDERS ONLY] Should patients have access to the prices charged by different providers for a medical procedure so they can shop around? (YES/NO)

Q13. Which comes closest to what you think about having an annual physical exam to monitor your health?

1. An annual physical exam is necessary
2. I wonder whether there are better alternatives for monitoring my health
3. An annual physical is unnecessary

Q14. How concerned are you about your exposure to radiation from tests such as x-rays, mammograms, angiograms, etc.?

1- Not at all concerned
2- Very Concerned

Q15. Please select the answer that comes closest to how you feel about new technology.

1. It is exciting and I use it as much as I can
2. It must be mastered to remain up-to-date
3. It's a bit beyond me
4. It scares me
