# Supplementary material for: How Consumers and Physicians View New Medical Technology: Comparative Survey
Source: J Med Internet Res. 2015 Sep 14;17(9):e215. doi: 10.2196/jmir.4456 (PMC4642377; doi:10.2196/jmir.4456)
Supplement: Multimedia Appendix 2 [file jmir_v17i9e215_app2.pdf]

2. Survey Items (Provider Survey Version)

Q1. Suppose medical technology advanced to the point where patients could do a test themselves and receive a diagnosis for non-life-threatening medical conditions. Which of the following statements best reflects your attitude towards patients' use of this kind of technology?

1. Patients should be able use new technology to diagnose non-life-threatening conditions
2. Patients should be able use new technology to get tests done, but diagnosis of any condition should be made by a qualified healthcare professional
3. I am uneasy about patients having access to new technology to diagnose anything, and feel that an in-person examination/diagnosis by a qualified healthcare professional is best

Q2. Do you support the use of genetic testing for each of the following medical situations? (YES/NO)

1. When planning to have a baby
2. To diagnose problems in a fetus
3. To identify and treat diseases (e.g. cancer)
4. For disease prevention
5. To identify and treat infections
6. To identify drug side effects
7. To prolong lifespan
8. To identify cause of death

Q3. In the future, it may be possible for your patients to perform blood tests using their smartphones. Would you be willing to accept blood tests via smartphones? (YES/NO)

Q4. Would you be willing to accept information from your patients' smartphones in place of an office visit for any of the following:
(YES/NO)

1. Suspicious skin problem
2. Heart rate and rhythm
3. Eye examination
4. Ear examination

Q5. Is the following statement true for you or not?
Because of concerns about privacy and security, I am hesitant to use digital health technology, such as storing, accessing, or sharing health records online and communicating with patients and other healthcare professionals electronically.
(TRUE FOR ME/NOT TRUE FOR ME)

Q6. Who owns your patients' medical records?

1. I own them
2. My patients own them
3. Don't know

Q7. Which of the following best represents your view toward patient access to medical records?

1. Lab and diagnostic tests results (X-rays, etc.)
    a. The patient has a right to see all of them
    b. I should share with patients only what I think is appropriate
2. Notes the doctor writes after visits or procedures
    a. The patient has a right to see all of them
    b. I should share with patients only what I think is appropriate

Q8. Do you believe that giving patients access to their detailed electronic health records could lead them to any of the following? (YES/NO)
1. Feeling anxious about the results
2. Better management of my health
3. Requesting unnecessary medical evaluations

Q9. Regarding lab test results, which do you think is the most appropriate?
1. Patients should have access to <u>all</u> their test results immediately
2. Healthcare professionals should review <u>all</u> test results before sharing with patients in case a discussion is needed
3. Healthcare professionals should review <u>only the types of test results that can cause patients to worry or panic</u> before sharing with patients

Q10. Do your patients tend to ask you questions about the cost of medical services prior to pursuing a course of treatment? (YES/NO)

Q11. Patients should have the right to know the full cost of a medical procedure before they decide whether to have it? (AGREE/DISAGREE)

Q12. Should patients have access to the prices charged by different providers for a medical procedure so they can shop around? (YES/NO)

Q12a. [PROVIDERS ONLY] If patients can shop around for medical care based upon price, are you prepared to compete on the basis of price? (YES/NO)

Q13. Which comes closest to what you think about annual physicals as a way to monitor patients' health?
1. An annual physical exam is necessary
2. There are better alternatives to the annual physical for monitoring patient health
3. An annual physical is unnecessary

Q14. How concerned are you about your patients' exposure to radiation when having such tests as x-rays, mammograms, angiograms, etc.?

1-Not at all concerned
2-Very Concerned

Q15. Please select the answer that comes closest to how you feel about new technology.
1. It is exciting and I use it as much as I can
2. It must be mastered to remain up-to-date
3. It's a bit beyond me
4. It scares me
